# Effects of Robot-Assisted Gait Training on Stage-Based Lower Limb Motor Recovery and Muscle Tone in Subacute Stroke: A Randomized Controlled Trial

**DOI:** 10.3390/jcm15072514

**Published:** 2026-03-25

**Authors:** Yoo Kyeong Han, Kyung Han Kim, Jung Eun Son, Arum Jeon, Hyo Been Lee, Miae Lee, Seong Gue Noh, Eo Jin Park, Seung Ah Lee, Sung Joon Chung, Dong Hwan Kim, Seung Don Yoo

**Affiliations:** 1 Department of Rehabilitation Medicine, Kyung Hee University Hospital at Gangdong, Seoul 05278, Republic of Korea; ykhan525@naver.com (Y.K.H.); rudgks421@naver.com (K.H.K.); gwendolen007@naver.com (J.E.S.); my6042@naver.com (A.J.); imiblg@khnmc.or.kr (M.L.); sgnsg000@khnmc.or.kr (S.G.N.); cp1024@naver.com (E.J.P.); lsarang80@gmail.com (S.A.L.); sungjoon.chung@gmail.com (S.J.C.); kdhkjr@paran.com (D.H.K.); 2 Healthcare Big-Data Center, Medical Science Research Institute, Kyung Hee University Hospital at Gangdong, Seoul 05278, Republic of Korea; 2hbhbhb2@naver.com

**Keywords:** subacute stroke, robot-assisted gait training, R-BoT Plus, Brunnstrom recovery stage, functional ambulation category, muscle tone, spasticity, MyotonPRO, stroke rehabilitation

## Abstract

**Background/Objectives:** Abnormal muscle tone and impaired motor control commonly limit gait recovery after stroke. Robot-assisted gait training has been introduced to augment conventional rehabilitation; however, its effects on stage-based motor recovery, functional ambulation, and muscle tone during the subacute phase remain unclear. **Methods:** This prospective, single-center, randomized controlled trial enrolled 30 patients with subacute stroke who received robot-assisted gait training plus conventional rehabilitation (R-BoT Plus group, *n* = 15) or conventional rehabilitation alone (control group, *n* = 15) over 4 weeks. The primary outcome was the change in Brunnstrom recovery stage of the lower extremities (BRS-LE). Secondary outcomes included Functional Ambulation Category (FAC), Fugl–Meyer Assessment for the Lower Extremity (FMA-LE), clinical spasticity measures (Modified Ashworth Scale and Modified Tardieu Scale), and muscle mechanical properties (MyotonPRO). Exploratory analyses were conducted to examine the associations between changes in stage-based motor recovery (ΔBRS-LE), functional ambulation (ΔFAC), and MyotonPRO parameters. Within-group changes were assessed using the Wilcoxon signed-rank test. Between-group effects were primarily evaluated using baseline-adjusted ANCOVA with HC3 robust standard errors, with Wilcoxon rank-sum tests on change scores as sensitivity analyses. Associations between changes in clinical outcomes and MyotonPRO parameters were evaluated using Spearman’s rank correlation coefficient (ρ). **Results:** BRS-LE (*p* = 0.014) and functional ambulation (*p* = 0.041) were significantly improved in the R-BoT Plus group. Changes in FMA-LE and clinical spasticity measures did not differ significantly between groups. Quantitative myotonometry revealed selective muscle- and parameter-specific changes. No robust correlations were observed between MyotonPRO parameters and changes in BRS-LE. **Conclusions:** The addition of robot-assisted gait training to conventional rehabilitation was associated with greater improvements in stage-based lower-limb motor recovery and functional ambulation in patients with subacute stroke. In contrast, cumulative impairment scores and conventional clinical spasticity measures demonstrated limited changes between groups. Quantitative muscle mechanical assessment revealed selective muscle-specific adaptations, supporting its role as a complementary tool for mechanistic characterization rather than as a surrogate marker of motor recovery. Future studies incorporating dose-matched designs and longer follow-up periods are warranted to clarify the independent and long-term effects of robot-assisted gait training.

## 1. Introduction

Stroke frequently results in impaired motor control of the lower extremities, leading to substantial limitations in gait, balance, and activities of daily living [1]. These impairments arise from multiple neuromuscular factors, including abnormal muscle activation, disrupted motor coordination, and altered muscle tone or spasticity [2,3]. Such changes may interfere with selective voluntary movement and contribute to maladaptive gait patterns, such as stiff-knee gait or equinovarus foot deformity [4]. Conventional physiotherapy and task-specific gait training remain the cornerstones of stroke rehabilitation; however, recovery of coordinated lower-limb movement is often incomplete, particularly during the early phases after stroke [5].

Robot-assisted gait training has emerged as a promising adjunct to conventional rehabilitation in patients with limited ambulatory capacity after stroke. By providing body weight support, repetitive stepping, and graded assistance, robotic devices allow the delivery of high-intensity task-specific gait practice in a safe and consistent manner [6]. Such training is thought to facilitate activity-dependent neuroplasticity, promote more physiological gait patterns, and support the recovery of coordinated lower limb movement through repeated activation of the neural and musculoskeletal systems [7].

Most previous studies of robot-assisted gait training have primarily focused on functional walking outcomes, such as gait speed, walking endurance, or ambulation level. Although these outcomes are clinically meaningful, they mainly reflect performance capacity rather than the underlying organization of motor control. Consequently, improvements in functional walking ability may not fully capture intervention-related changes in the structure of motor recovery during the early phases after stroke [2].

Motor recovery after stroke involves hierarchical reorganization of motor control, progressing from flaccidity and synergistic movement patterns toward increasingly selective voluntary motor coordination [8]. The Brunnstrom recovery stage for the lower extremities (BRS-LE) describes this evolution and is widely used in clinical practice. In contrast to cumulative motor scales such as the Fugl–Meyer Assessment for the Lower Extremity (FMA-LE), which quantify impairment using summed task-performance scores, the BRS-LE classifies recovery into discrete hierarchical stages that reflect qualitative changes in motor organization. Thus, while the FMA-LE captures incremental improvements in motor performance, the BRS-LE reflects transitions in motor coordination and the resolution of abnormal synergies. Consequently, progression in the BRS-LE represents transitions in motor coordination rather than incremental increases in strength or joint movement.

During the subacute phase after stroke, motor recovery is characterized by rapid neural reorganization and evolving motor strategies [9]. In this context, stage-based measures such as the BRS-LE may be particularly sensitive to early changes in motor coordination that may not yet produce substantial changes in cumulative impairment scores [10]. Robot-assisted gait training delivers repetitive, task-specific stepping under partially normalized kinematic conditions with graded assistance. Such structured sensorimotor practice may facilitate activity-dependent plasticity within descending motor pathways and spinal locomotor networks [11], potentially promoting the resolution of abnormal lower-limb synergies. Therefore, evaluating stage-based motor recovery using the BRS-LE may provide insight into whether robot-assisted gait training influences the organization of voluntary movement rather than only functional walking performance.

Functional ambulation is a key clinical manifestation of lower limb motor recovery during inpatient rehabilitation. The Functional Ambulation Category (FAC) provides a pragmatic classification of walking ability based on the level of physical assistance required and reflects activity-level ambulation in routine clinical settings [12]. The Fugl–Meyer Assessment for the Lower Extremity (FMA-LE) is a validated measure of lower limb motor impairment after stroke [13]. In this study, the FMA-LE was included as a secondary outcome to complement the primary stage-based assessment (BRS-LE) by providing quantitative information on impairment-level motor performance.

However, evidence regarding the effects of robot-assisted gait training on muscle tone remains inconsistent. One possible explanation is the reliance on conventional clinical scales, such as the Modified Ashworth Scale (MAS) and Modified Tardieu scale (MTS), which have limited sensitivity to subtle or short-term changes in muscle mechanical properties [14]. These limitations have led to increasing interest in quantitative approaches for assessing muscle mechanical characteristics.

In addition to clinical scales, quantitative tools have been developed to objectively assess the mechanical properties of muscles. MyotonPRO is a handheld myometric device that allows for the noninvasive measurement of muscle oscillation frequency, stiffness, logarithmic decrement, relaxation time, and creep, reflecting the viscoelastic properties of muscle tissue [15]. These parameters are not intended to replace functional outcome measures but may provide complementary, muscle-specific insight into mechanical behavior that is not captured by ordinal spasticity scales [16]. Importantly, during the subacute phase after stroke, improvements in motor control and functional performance may precede measurable normalization of passive muscle mechanical properties, suggesting a dissociation between motor recovery and changes in muscle stiffness [3,17].

This randomized controlled trial investigated whether an augmented rehabilitation program incorporating robot-assisted gait training (R-BoT Plus) enhances lower limb motor recovery in patients with subacute stroke and gait disturbances. The BRS-LE was selected as the primary outcome to evaluate stage-based lower-limb motor recovery and motor organization. Functional ambulation (FAC) and impairment-level motor performance (FMA-LE) were included as secondary outcomes, while muscle tone was assessed using clinical scales (MAS, MTS) and quantitative muscle mechanical measurements (MyotonPRO).

The aims of this study were to:

1. Determine whether adding robot-assisted gait improves stage-based lower limb motor recovery, as assessed by the BRS-LE (primary outcome).
2. Evaluate changes in functional ambulation (FAC) and assess supportive changes in lower limb motor impairment (FMA-LE).
3. Assess changes in muscle tone using the MAS, MTS, and MyotonPRO, and explore their association with changes in stage-based lower limb motor recovery (ΔBRS-LE).

We hypothesized that the addition of robot-assisted gait training to conventional rehabilitation would result in greater improvement in stage-based motor recovery, as reflected by changes in the BRS-LE. We further hypothesized that improvements in BRS-LE would be accompanied by gains in functional ambulation (FAC), whereas changes in FMA-LE and passive muscle mechanical properties might show more modest or heterogeneous changes over the intervention period.

## 2. Materials and Methods

### 2.1. Study Design and Setting

This was a prospective, single-center, randomized controlled trial investigating the effects of robot-assisted gait training on lower limb motor recovery in patients with subacute stroke, with an exploratory evaluation of muscle tone-related characteristics. The participants were consecutively recruited from the inpatient rehabilitation unit of a university-affiliated tertiary hospital.

The study protocol was approved by the Institutional Review Board of Kyung Hee University Hospital at Gangdong, Korea (KHNMC IRB 2024-11-025), and all procedures were conducted in accordance with the Declaration of Helsinki. Written informed consent was obtained from all participants or their legally authorized representatives before enrollment.

### 2.2. Participants

Eligible participants were adults aged 20 years or older who met the following criteria: (1) first-ever or recurrent ischemic or hemorrhagic stroke confirmed by neuroimaging; (2) subacute stage of stroke, defined as 1 week to 3 months after stroke onset; (3) impaired lower limb motor function with limited independent ambulation at baseline, defined as a FAC score of 2 or lower; and (4) ability to follow simple instructions and participate in rehabilitation sessions.

Exclusion criteria included: (1) contraindications to robot-assisted gait training or body-weight-supported stepping, such as severe cardiovascular instability or uncontrolled arrhythmia; (2) severe musculoskeletal disorders or fixed lower limb contractures limiting safe weight-bearing or stepping; (3) co-existing neurological disorders affecting gait (e.g., Parkinson’s disease or advanced peripheral neuropathy); (4) severe cognitive or communication impairments precluding reliable participation or outcome assessment; and (5) other medical conditions judged by the investigators to pose excessive risk during the intervention.

### 2.3. Interventions

#### 2.3.1. Robot-Assisted Gait Training Group (R-BoT Plus Group)

**Figure 1:**
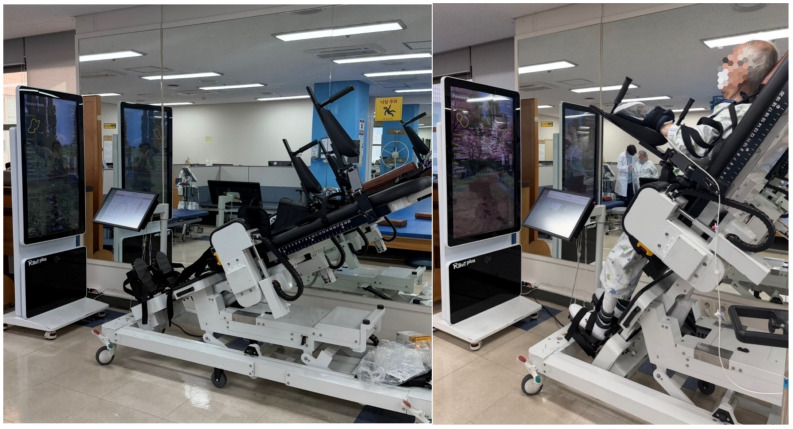
R-BoT Plus system (CoTras, Daegu, Republic of Korea).

The participants allocated to the experimental group received robot-assisted gait training using the R-BoT Plus system (CoTras, Daegu, Republic of Korea), which is a robotic device designed to facilitate early gait-related training through body-weight-supported guided stepping (Figure 1). The training parameters, including body weight support, stepping speed, guidance force, and table tilt angle, were individually adjusted according to each participant’s functional level and tolerance.

The system allows the optional application of functional electrical stimulation to selected lower limb muscles and provides real-time visual feedback via a monitoring interface to enhance active participation and motor learning. The participants received approximately 30 min of robot-assisted gait training, in addition to 30 min of conventional gait and lower limb rehabilitation per session, five times per week for 4 weeks. All sessions were supervised by experienced physical therapists to ensure safety and appropriate adjustment of the training parameters.

#### 2.3.2. Control Group

Participants in the control group received conventional gait and lower limb rehabilitation only for approximately 30 min per session, five times per week, for 4 weeks. Conventional therapy includes overground gait training, balance-related tasks, lower limb strengthening, and functional task-oriented exercises. The content and frequency of conventional therapy were comparable to those provided to the experimental group except for the absence of robot-assisted gait training.

### 2.4. Outcome Measures

All outcome measures were assessed at baseline (pre-intervention) and within 3 days of completion of the 4-week intervention period (post-intervention).

#### 2.4.1. Primary Outcome—Brunnstrom Recovery Stage for the Lower Extremity

The BRS-LE describes the progression of motor recovery from flaccidity through spastic synergy patterns and increasingly selective voluntary movements. Higher stages indicated more advanced motor recovery and better selective motor control. The BRS-LE was defined as the primary outcome, and the primary endpoint was the change in BRS-LE from baseline to post-intervention.

#### 2.4.2. Secondary Outcomes—Functional and Motor Measures

Functional walking ability was assessed using the FAC, a six-level ordinal scale that classifies ambulation according to the degree of physical assistance required, ranging from non-functional ambulation to independent walking on uneven surfaces. The FAC was used as a secondary functional outcome to assess changes in activity-level walking ability during the intervention period.

Lower limb motor performance was assessed using the Korean version of the FMA-LE. In this study, the total FMA-LE score, comprising motor and coordination components (total score range, 0–34), was used to quantify changes in lower limb motor impairment.

#### 2.4.3. Secondary Outcomes—Muscle Tone and Biomechanical Properties

Muscle tone was assessed using both clinical scales and quantitative measurements.

Clinical spasticity was evaluated using the MAS and MTS. The MAS grades resistance to passive stretching on an ordinal scale and was evaluated in selected upper and lower limb muscle groups, including the elbow flexors, wrist flexors, hip adductors, knee extensors, and ankle plantar flexors.

Dynamic spasticity was assessed using the MTS, which quantifies velocity-dependent resistance to passive stretch and was evaluated at multiple joint muscle groups, including the elbow flexors, elbow extensors, wrist flexors, wrist extensors, hip abductors, hip adductors, knee flexors, knee extensors, ankle dorsiflexors, and ankle plantarflexors. For each joint, the angle of muscle reaction during slow (R2) and fast (R1) passive stretching was measured, and the R2–R1 difference was calculated as an index of dynamic resistance.

The quantitative muscle mechanical properties were assessed using a handheld myotonometer (MyotonPRO; Myoton AS, Tallinn, Estonia), which provides objective measurements of muscle viscoelastic properties. The device applies a brief mechanical impulse under constant prepressure (0.18 N) to induce natural oscillations of the underlying tissue. The following parameters were derived: oscillation frequency (Hz), dynamic stiffness (N/m), logarithmic decrement, relaxation time (ms), and creep.

Measurements were performed bilaterally on both the paretic (PS) and the non-paretic (N-PS) sides in gait-relevant muscles, including the rectus femoris, tibialis anterior, biceps femoris, and medial gastrocnemius. Measurement sites were identified using predefined anatomical landmarks to ensure reproducibility. Specifically, the rectus femoris was measured at the distal two-thirds point between the anterior superior iliac spine and the superior border of the patella; the tibialis anterior at the proximal one-third between the medial malleolus and the fibular head (approximately 2 cm lateral to the tibial crest); the biceps femoris at the proximal two-thirds point between the ischial tuberosity and the popliteal crease; the medial gastrocnemius at the proximal one-third between the medial malleolus and the popliteal crease.

All assessments were conducted under passive resting conditions in standardized positions. Participants rested for at least 5 min prior to measurement. For each muscle, three consecutive measurements were obtained and averaged for analysis. All measurements were performed by a single trained examiner to ensure procedural consistency. Change scores were calculated as post-intervention minus baseline values.

### 2.5. Statistical Analysis

Baseline characteristics are summarized as mean ± standard deviation for continuous variables and frequencies with percentages (%) for categorical variables. Group comparability was assessed using the chi-squared test or Fisher’s exact test for categorical data. For continuous variables, normality was evaluated using the Shapiro–Wilk test. The independent t-test or Wilcoxon rank-sum test was used for normally and non-normally distributed data, respectively. To ensure consistency with prior rehabilitation literature and enhance clinical interpretability, ordinal scales were summarized as mean ± SD.

Within-group changes from baseline to post-treatment were analyzed using the Wilcoxon signed-rank test. To evaluate the efficacy of the intervention, individual-level change scores were calculated as Δ = Post − Pre. For these within-group analyses, the Hodges–Lehmann estimator for paired differences and their corresponding 95% confidence intervals (CIs) were reported.

The primary between-group treatment effect was estimated using analysis of covariance (ANCOVA) to obtain baseline-adjusted group differences. In this model, the post-treatment score was specified as the outcome, with the baseline (pre-treatment) score included as a covariate and group assignment as the main predictor. To account for potential violations of model assumptions, HC3 heteroscedasticity-robust standard errors were used to derive 95% CIs and *p*-values for the adjusted group differences. The magnitude of the treatment effect was quantified using Hedges’ *g*. As sensitivity analyses, nonparametric between-group comparisons of change scores were conducted using the Wilcoxon rank-sum test, with treatment effects summarized using the Hodges–Lehmann estimator.

Spearman’s rank correlation coefficient (ρ) was used to explore the relationship between changes in stage-based motor recovery (ΔBRS-LE) and functional ambulation (ΔFAC). Associations between changes in clinical outcomes and changes in muscle mechanical properties (MyotonPRO parameters) were also evaluated using Spearman’s correlation. To account for hemiparesis-related clinical differences, MyotonPRO data initially recorded for anatomical side (left or right) were reclassified as PS and N-PS based on individual clinical presentation. Correlation analyses were performed for the total cohort and stratified by clinical classification (PS vs. N-PS) to examine side-specific relationships.

All analyses were performed using R (version 4.2.3; R Foundation for Statistical Computing, Vienna, Austria). All statistical tests were two-sided, and *p* < 0.05 was considered statistically significant. Additional analyses, including sensitivity analyses, are provided in the Supplementary Materials.

## 3. Results

### 3.1. Participant Characteristics

**Figure 2:**
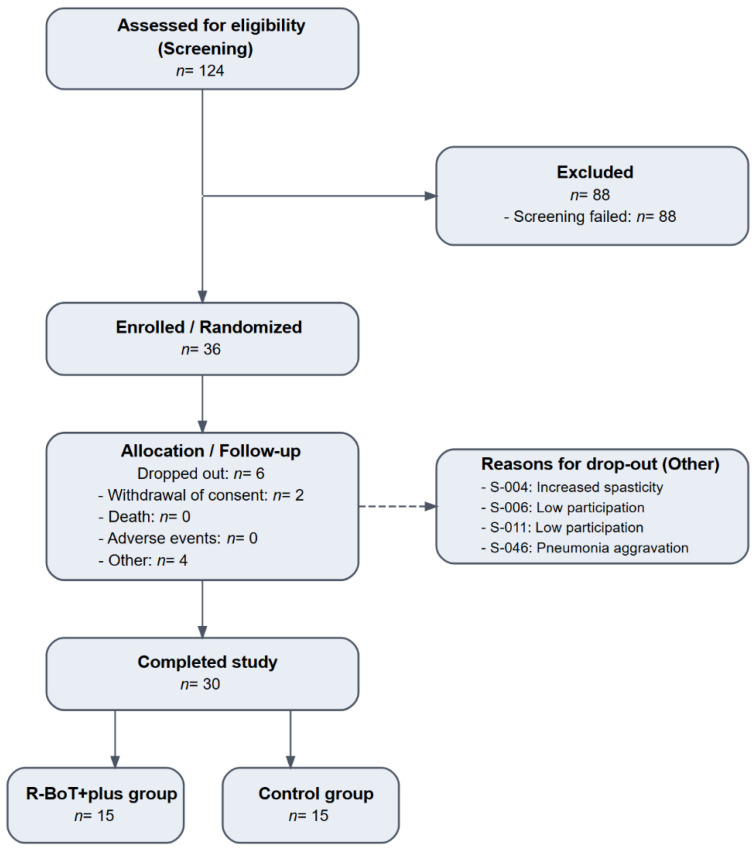
Flowchart diagram of the study participants.

Thirty participants with subacute stroke were enrolled and randomized to the robot-assisted gait training group (R-BoT Plus group, *n* = 15) or control group (*n* = 15) (Figure 2). Baseline demographic and clinical characteristics of the participants are shown in Table 1. There were no significant between-group differences at baseline with respect to age, sex distribution, stroke type, time since stroke onset, stage-based lower limb motor recovery assessed using the BRS-LE, lower limb motor impairment measured using the FMA-LE, or functional ambulation status assessed using the FAC (all *p* > 0.05). No serious adverse events related to the intervention were observed during the study period.

### 3.2. Primary Outcome: Brunnstrom Recovery Stage for the Lower Extremity

**Figure 3:**
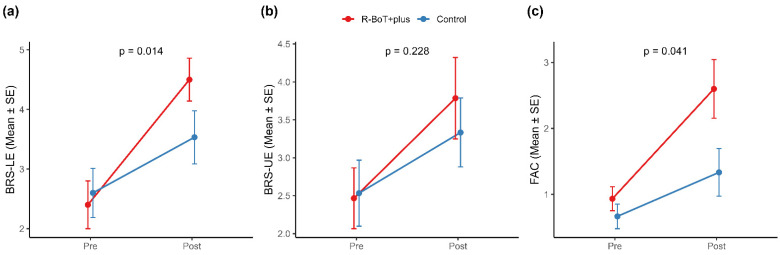
Changes in stage-based motor recovery and functional ambulation following the intervention. Mean (± SE) scores for the BRS-LE (**a**), BRS-UE (**b**), and Functional Ambulation Category (**c**) from baseline to post-intervention. The *p*-values indicate baseline-adjusted between-group differences estimated using ANCOVA with HC3 heteroscedasticity-robust standard errors. BRS-LE, Brunnstrom Recovery Stage for the lower extremities; BRS-UE, Brunnstrom Recovery Stage for the upper extremities; FAC, Functional Ambulation Category; SE, standard error.

Changes in the BRS-LE over the 4-week intervention period are summarized in Table 2 and illustrated in Figure 3. Both the robot-assisted gait training and control groups demonstrated improvements in the BRS-LE from baseline to post-intervention, consistent with ongoing motor recovery during the subacute phase after stroke.

A greater proportion of the participants in the robot-assisted gait training group demonstrated an improvement in the recovery stage, with several individuals progressing through at least one stage of BRS-LE. Improvements were also observed in the control group. However, individual responses were more variable, with smaller or absent stage changes observed in some participants.

In the primary analysis using ANCOVA adjusted for baseline BRS-LE, the robot-assisted gait training group showed a significantly greater improvement than the control group (adjusted group difference, 1.09; 95% CI, 0.24 to 1.94; *p* = 0.014; Table 2). Sensitivity analysis using the Wilcoxon rank-sum test on change scores yielded consistent results (*p* = 0.004; Supplementary Table S2).

### 3.3. Secondary Motor and Functional Outcomes: FAC and FMA-LE

Secondary outcomes showed different inter-group effects on ambulatory function and motor performance (Table 2 and Figure 3). Functional ambulation assessed using the FAC demonstrated a significant between-group difference, favoring the robot-assisted gait training group (adjusted group difference, 0.81; 95% CI, 0.03 to 1.59; *p* = 0.041; Table 2). Sensitivity analysis using the Wilcoxon rank-sum test on change scores showed consistent findings (*p* = 0.035; Supplementary Table S2). Participants who received robot-assisted gait training showed greater improvement in walking assistance levels than those who received conventional rehabilitation alone, indicating a greater improvement in ambulatory independence over the intervention period.

An exploratory correlation analysis was conducted to examine the relationship between changes in stage-based motor recovery and functional ambulation. The change in BRS-LE was not significantly correlated with the change in FAC (Spearman ρ = −0.269, *p* = 0.352).

Lower limb motor performance, assessed using the FMA-LE, improved in both groups over the 4-week intervention period; however, between-group comparisons did not demonstrate a statistically significant difference (Supplementary Table S1). Changes in the FMA-LE scores varied across participants, without a consistent pattern favoring either intervention.

### 3.4. Clinical Muscle Tone Measures: Modified Ashworth Scale and Modified Tardieu Scale

Clinical spasticity outcomes assessed using the MAS are summarized in Supplementary Table S1. At baseline, the MAS scores varied across participants and muscle groups, reflecting heterogeneity in the severity of initial spasticity. Over the 4-week intervention period, the MAS scores exhibited only small numerical changes in both the robot-assisted gait training and control groups. These changes were inconsistent across individual muscles and participants without a uniform directional pattern or statistically significant between-group differences.

Dynamic spasticity assessed using the MTS demonstrated a similar profile (Supplementary Table S1). The baseline R2–R1 values differed across joints and participants, indicating heterogeneous velocity-dependent resistance at study entry. During the intervention period, changes in the R2–R1 values were modest and variable across joints, and no MTS parameter showed a statistically significant between-group difference or a consistent trend toward reduction in either group.

### 3.5. Quantitative Muscle Mechanical Properties: MyotonPRO

The quantitative muscle mechanical properties assessed using MyotonPRO are summarized in Table 3 and Supplementary Table S3, which present results for both PS and N-PS in the experimental and control groups. At baseline, the MyotonPRO parameters showed substantial inter-individual variability across muscles, reflecting heterogeneous passive muscle mechanical characteristics during the subacute phase after stroke.

Over the 4-week intervention period, changes in MyotonPRO-derived parameters were generally small and muscle-specific across both PS and N-PS in both groups. For most of the assessed muscles and parameters, between-group comparisons of change scores did not demonstrate statistically significant differences, and no uniform intervention-related effects on passive muscle mechanical properties were observed (Supplementary Table S3).

In contrast, in the PS biceps femoris, the between-group analysis revealed significant differences in selected relaxation-related parameters (Table 3). Compared to the control group, the robot-assisted gait training group demonstrated a greater increase in oscillation frequency and a greater decrease in relaxation time over the intervention period (both *p* < 0.05). Although MyotonPRO parameters were analyzed bilaterally, statistically significant between-group differences were observed only in selected parameters of the PS biceps femoris. No other MyotonPRO parameters showed statistically significant between-group differences for this muscle.

### 3.6. Associations Between Changes in Brunnstrom Recovery Stage for the Lower Extremities and MyotonPRO Parameters

**Figure 4:**
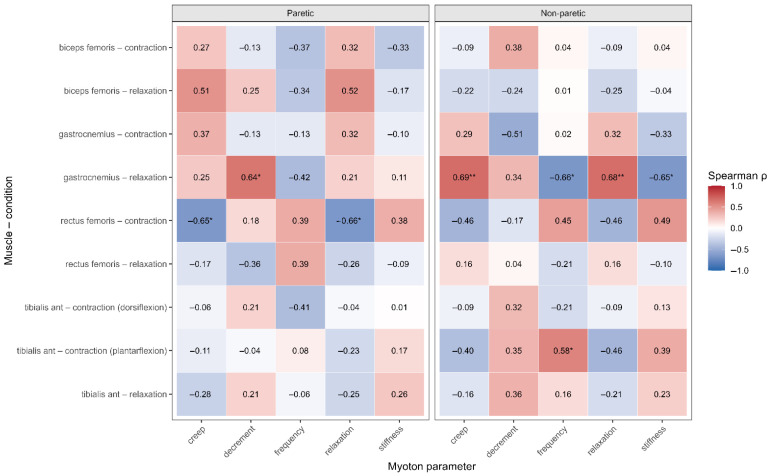
Heatmap illustrating exploratory correlations between changes in Δ Brunnstrom Recovery Stage for the lower extremities and changes in MyotonPRO parameters in gait-related muscles of the paretic limb. Spearman’s rank correlation coefficients (ρ) are shown for individual muscle–parameter pairs. Statistically significant correlations (*p* < 0.05) are indicated using asterisks. Significant correlations are denoted by asterisks (* *p* < 0.05, ** *p* < 0.01).

Exploratory analyses were conducted to examine associations between changes in stage-based lower limb motor recovery (ΔBRS-LE) and changes in quantitative muscle mechanical properties assessed using MyotonPRO (Supplementary Table S4, Figure 4). These analyses were restricted to gait-related muscles of the paretic side (PS), as the BRS-LE reflects stage-based motor recovery of the affected limb after stroke.

Statistically significant associations were limited and observed only in selected muscle–parameter pairs. In the rectus femoris, BRS-LE was significantly associated with changes in creep (ρ = −0.647, *p* = 0.012) and relaxation time (ρ = −0.657, *p* = 0.011), suggesting a relationship between stage-based motor recovery and viscoelastic muscle behavior in this proximal gait-related muscle. In the gastrocnemius, ΔBRS-LE was significantly correlated with the logarithmic decrement (ρ = 0.640, *p* = 0.014). No significant association was identified in the tibialis anterior.

## 4. Discussion

This randomized controlled trial examined the effects of robot-assisted gait training combined with conventional rehabilitation on lower limb motor recovery and muscle tone-related measures in patients with subacute stroke. The primary finding was a greater improvement in BRS-LE in the group receiving additional robotic training. Improvements in functional ambulation were also observed, whereas between-group differences in cumulative impairment scores and conventional spasticity scales were limited.

Importantly, most prior trials of robot-assisted gait training have focused primarily on functional walking outcomes, such as gait speed or ambulation level [6,11]. In contrast, the present study selected stage-based motor recovery as the primary endpoint to examine whether repetitive, high-intensity stepping practice might influence the organization of lower-limb motor control, in addition to improving functional walking performance. This distinction aligns with contemporary stroke recovery frameworks emphasizing the importance of targeting motor reorganization in addition to functional performance [18].

### 4.1. Motor Recovery After Stroke: Stage-Based Coordination Changes and Impairment-Level Motor Performance

The observed improvement in BRS-LE highlights the clinical relevance of stage-based motor assessment during the subacute phase after stroke, a period characterized by rapid neural reorganization and evolution of motor strategies [18,19]. In the present study, a significant between-group improvement was observed in BRS-LE, whereas no significant difference was identified in FMA-LE. This discrepancy provides important insight into the distinct aspects of motor recovery captured by these measures.

Although both BRS-LE and FMA-LE are considered impairment-level assessments, they differ fundamentally in their conceptual structure [20]. The BRS-LE reflects stage-based transitions in motor coordination, capturing qualitative changes such as the resolution of abnormal synergistic movement patterns and the emergence of selective voluntary control. In contrast, the FMA-LE represents a cumulative quantitative score based on task performance across multiple joint movements.

This difference in measurement properties may explain the observed divergence in responsiveness. During the subacute phase after stroke, motor recovery is often characterized by rapid reorganization of coordination patterns, whereas measurable improvements in joint-specific motor performance may occur more gradually [21,22]. As a result, relatively small but clinically meaningful improvements in movement selectivity may lead to progression in BRS-LE without producing sufficiently large changes in total FMA-LE scores over a short intervention period.

Taken together, these findings suggest that stage-based measures such as the BRS-LE may be more sensitive to early changes in motor organization, whereas cumulative impairment scales such as the FMA-LE may require longer observation periods to detect significant changes. Importantly, the absence of a between-group difference in FMA-LE does not contradict the observed improvement in stage-based motor recovery but rather reflects the complementary nature of these assessment tools.

The BRS-LE captures the progression of motor recovery through hierarchical stages, reflecting transitions from obligatory synergistic movement patterns toward increasingly selective voluntary control. These stage transitions represent qualitative shifts in motor coordination rather than incremental increases in strength or joint excursion [10].

Robot-assisted gait training delivers repetitive, task-specific stepping under partially normalized kinematic conditions with graded assistance. From a neurophysiological perspective, structured and high-frequency sensorimotor practice may facilitate activity-dependent plasticity within descending motor pathways and spinal locomotor networks [11]. Repeated exposure to more physiological stepping patterns could support the resolution of abnormal lower-limb synergies and the emergence of more selective control [22]. In this context, progression in BRS-LE may reflect reorganization of motor coordination rather than merely enhanced performance output.

By designating BRS-LE as the primary outcome, this study aimed to evaluate whether robot-assisted gait training might influence this hierarchical aspect of motor recovery. The observed stage progression therefore provides evidence that augmented gait practice may affect the organization of voluntary movement during the subacute phase. These stage-based improvements may precede or occur independently of detectable changes in cumulative impairment scores, while still contributing to functional gains in walking ability, as reflected by improvements in the Functional Ambulation Category (FAC).

### 4.2. Functional Ambulation

Functional ambulation assessed using the FAC also improved in the experimental group. The FAC captures activity-level walking independence and has been shown to be sensitive to clinically meaningful changes during inpatient stroke rehabilitation [12]. These findings suggest that the addition of robot-assisted gait training may contribute to improvements in ambulatory independence during the subacute phase after stroke.

Descriptive examination of the data indicated that all participants who demonstrated improvement in FAC (ΔFAC ≥ 1) also showed concurrent improvement in BRS-LE (ΔBRS-LE ≥ 1). This observation suggests that improvements in lower-limb motor recovery may accompany gains in functional ambulation in this cohort.

However, exploratory correlation analysis did not demonstrate a statistically significant association between the magnitude of change in BRS-LE and FAC. This finding indicates that although improvement in FAC was observed only among participants who also improved in BRS-LE, the extent of change in the two measures did not show a clear linear relationship at the individual level.

One possible explanation is that the two measures capture different aspects of recovery. While BRS-LE reflects impairment-level motor recovery of the lower limb, the FAC represents activity-level walking independence, which may also be influenced by balance control, compensatory strategies, assistive support, and environmental factors. In addition, the exploratory nature of this analysis and the modest sample size may have limited the ability to detect a stable association between the two measures. Future studies with larger cohorts may help clarify the relationship between lower-limb motor recovery and functional ambulation during stroke rehabilitation.

### 4.3. Muscle Tone and Neuromuscular Properties in Stroke Rehabilitation

Changes in muscle tone, assessed using the MAS and the MTS, were small and heterogeneous. Baseline MAS grades varied widely across participants, reflecting variability in the severity of initial spasticity. Such heterogeneity may reduce the sensitivity and responsiveness of ordinal spasticity scales, particularly when applied to heterogeneous subacute stroke populations [14,23]. Moreover, the MAS and MTS primarily assess resistance to passive stretching under non-functional conditions. In the early and subacute phases after stroke, increased muscle tone is not uniformly maladaptive; in some patients, residual spasticity may transiently contribute to postural stability or facilitate task-specific muscle activation during rehabilitation [17]. Therefore, changes in passive resistance measured using the MAS or MTS may not necessarily correspond to improvements in active motor control or gait performance.

Complementing these clinical scales, quantitative assessment of muscle mechanical properties using MyotonPRO provides additional insight into the mechanical behavior of specific muscles. In the present study, MyotonPRO revealed selective changes in specific muscle–parameter combinations, most notably in relaxation-related parameters of the biceps femoris. The biceps femoris plays a pivotal role in gait control particularly during terminal swing and early stance, where it contributes to knee flexion, deceleration of forward tibial progression, and stabilization of the knee prior to heel strike [24,25]. In individuals with stroke, impaired modulation of hamstring activity has been associated with altered swing phase initiation, reduced knee flexion, and compensatory gait patterns such as stiff-knee gait [26,27,28].

These findings suggest that repetitive, task-specific gait training may induce localized neuromechanical adaptations in muscles directly involved in locomotor control rather than producing a uniform reduction in muscle tone across the lower limb. Such muscle-specific responses are consistent with contemporary precision-based approaches to spasticity management, which recognize that spastic muscle behavior varies according to anatomical structure and functional role during movement [29,30]. Similarly, recent ultrasound-guided botulinum toxin injection frameworks emphasize targeting anatomically and functionally relevant regions within specific gait-related muscles to optimize treatment outcomes [29,30]. In this context, differential changes in parameters such as relaxation time in the biceps femoris may reflect muscle-specific neuromechanical adaptation rather than global reductions in tone.

Importantly, robust correlations between MyotonPRO parameters and changes in BRS-LE were not observed. This dissociation highlights the complexity of neuromuscular adaptation during subacute recovery rather than undermining the relevance of quantitative muscle assessment. Motor recovery during the subacute phase may primarily reflect neural reorganization and improved coordination, which may occur without parallel normalization of passive muscle mechanical properties [23]. Taken together, these findings suggest that the neuromuscular mechanical properties and functional recovery may evolve through partially independent processes during the early stages of stroke rehabilitation.

From a broader conceptual perspective, these findings can be interpreted within the framework of the International Classification of Functioning, Disability and Health (ICF). Within this model, muscle tone and mechanical muscle properties represent impairment-level characteristics, whereas walking ability and ambulation reflect activity-level outcomes [31,32]. Accordingly, impairment-level neuromuscular adaptations may not always translate directly into immediate changes in functional performance during early stroke recovery.

In line with this framework, quantitative assessment of muscle mechanical properties should be interpreted as providing mechanistic insight into impairment-level neuromuscular behavior rather than directly representing functional recovery. Quantitative myotonometry should therefore not be viewed as a surrogate outcome for motor recovery but rather as a mechanistic characterization tool that complements clinical and functional assessments. In rehabilitation research, such measurements may help identify muscle-specific adaptations associated with gait training, while overall functional outcomes remain primarily determined by broader neural reorganization and improvements in motor coordination.

The selective pattern of changes observed in this study underscores the importance of hypothesis-driven evaluation of specific muscles and mechanical parameters within clearly defined functional contexts.

### 4.4. Limitations

This study had several limitations. First, the total therapy time differed between groups, as the experimental group received additional robot-assisted gait training alongside conventional rehabilitation. Therefore, the observed effects may partly reflect increased therapy intensity rather than the isolated effect of the robotic intervention.

Second, the sample size was modest, which may have limited the statistical power to detect smaller between-group differences, particularly in secondary and exploratory outcomes.

Third, variability in baseline spasticity severity may have reduced the responsiveness of ordinal clinical scales such as the MAS and MTS, potentially contributing to the absence of clear group differences.

Fourth, muscle mechanical properties were assessed under passive resting conditions and may not fully reflect dynamic muscle behavior during functional activities such as gait.

Finally, this study did not include follow-up assessments after the intervention period. As a result, the long-term sustainability of the observed motor recovery and neuromuscular adaptations could not be evaluated.

### 4.5. Strengths and Implications for Future Research

Despite these limitations, this study had several strengths. The randomized controlled design and focus on stage-based motor recovery allowed for the detection of clinically meaningful changes relevant to gait rehabilitation in patients with subacute stroke. The combined use of BRS-LE and FAC enabled differentiation between motor organization and functional ambulation, providing a nuanced understanding of recovery beyond impairment level scores alone.

Importantly, the integration of quantitative muscle mechanical assessments with conventional spasticity scales highlights an emerging methodological direction for spasticity research. Rather than relying solely on ordinary clinical scales, future studies may benefit from the incorporation of objective muscle-specific measurements to explore the mechanisms of neuromuscular adaptation. Stratification by baseline spasticity severity and hypothesis-driven selection of muscles and parameters may further enhance the interpretability of these findings.

In addition, future trials incorporating dose-matched control conditions are warranted to clarify the independent contribution of robotic assistance beyond overall therapy intensity. Future studies should also incorporate longer follow-up assessments to determine whether the observed improvements in stage-based motor recovery and functional ambulation translate into sustained functional gains over time.

## 5. Conclusions

In patients with subacute stroke, the addition of robot-assisted gait training to conventional rehabilitation resulted in greater improvements in stage-based lower-limb motor recovery and functional ambulation. The observed improvement in BRS-LE suggests enhanced motor coordination and reorganization during the subacute phase. In contrast, cumulative impairment scores and conventional spasticity measures showed limited between-group differences. Quantitative myotonometry revealed selective, muscle-specific changes, supporting its role as a complementary tool for mechanistic assessment rather than a surrogate marker of motor recovery.

Future studies incorporating dose-matched designs and longer follow-up periods are warranted to clarify the independent and long-term effects of robot-assisted gait training.

## Figures and Tables

**Table 1:** Baseline characteristics.

Characteristics	R-BoT Plus	Control	*p*-Value
	(*n* = 15)	(*n* = 15)	
**Demographics**			
Sex			0.699
Male	11 (73.33%)	9 (60%)	
Female	4 (26.67%)	6 (40%)	
Age (years)	64.80 ± 12.67	66.20 ± 13.93	0.776
Height (cm)	167.40 ± 7.08	167.07 ± 9.20	0.912
Weight (kg)	73.93 ± 10.45	68.53 ± 16.87	0.070
BMI (kg/m^2^)	26.30 ± 2.60	24.42 ± 5.14	0.021
Time since stroke (days)	21.53 ± 9.35	26.13 ± 11.48	0.239
Lesion type			1.000
Hemorrhagic	4 (26.67%)	3 (20%)	
Ischemic	11 (73.33%)	12 (80%)	
Hemiplegic side			1.000
Left	9 (60%)	10 (66.67%)	
Right	6 (40%)	5 (33.33%)	
**Functional Scores**			
BRS-UE	2.47 ± 1.55	2.53 ± 1.68	1.000
BRS-LE	2.40 ± 1.55	2.60 ± 1.59	0.931
FMA-LE	17.40 ± 8.64	14.80 ± 13.08	0.633
FAC, median (IQR)	1.00 (0.50, 1.00)	1.00 (0.00, 1.00)	0.299

BRS-LE, Brunnstrom recovery stage for the lower extremities; BRS-UE, Brunnstrom recovery stage for the upper extremities; FAC, Functional Ambulation Category; FMA-LE, Fugl–Meyer Assessment for the Lower Extremity; IQR, interquartile range.

**Table 2:** Pre- and post-treatment scores and baseline-adjusted between-group differences in motor recovery and functional ambulation outcomes.

Outcome	Pre	Post	Adjusted Group Difference ^†^ (95% CI)	*p*-Value	Effect Size (Hedges’ g) ^‡^
BRS-LE					
R-BoT + plus	2.43 ± 1.60	4.50 ± 1.34	1.09 (0.24, 1.94)	0.014	0.99
Control	2.60 ± 1.59	3.53 ± 1.73			
BRS-UE					
R-BoT + plus	2.36 ± 1.55	3.79 ± 2.01	0.61 (−0.40, 1.62)	0.228	0.49
Control	2.53 ± 1.68	3.33 ± 1.76			
FAC					
R-BoT + plus	0.93 ± 0.70	2.60 ± 1.72	0.81 (0.03, 1.59)	0.041	0.87
Control	0.67 ± 0.72	1.33 ± 1.40			

BRS-LE, Brunnstrom recovery stage for the lower extremities; BRS-UE, Brunnstrom recovery stage for the upper extremities; FAC, Functional Ambulation Category; CI, confidence interval; SD, standard deviation. Values are presented as mean ± SD. ^†^ Adjusted difference indicates the baseline-adjusted between-group difference (R-BoT + plus minus Control) estimated from ANCOVA with post-treatment score as the outcome, group as the factor of interest, and baseline score as a covariate. HC3 heteroscedasticity-robust standard errors were used to derive 95% CIs and *p*-values. ^‡^ Effect sizes were quantified using Hedges’ g based on change scores (Post–Pre).

**Table 3:** Comparison of changes in biceps femoris relaxation Myoton parameters from baseline to post-treatment between groups, stratified by paretic status (PS vs. N-PS).

Outcome	PS		N-PS	
	R-BoT Plus	Control	R-BoT Plus	Control
Frequency				
Mean change (SD)	1.26 ± 2.27	−0.65 ± 2.05	0.42 ± 2.52	−9.27 ± 38.34
Group difference (95% CI)	1.91 (0.29, 3.53)		9.69 (−11.57, 30.96)	
*p*-value	0.020		0.709	
Relaxation				
Mean change (SD)	−1.55 ± 4.52	1.75 ± 4.79	0.69 ± 7.91	−16.52 ± 58.42
Group difference (95% CI)	−3.31 (−6.79, 0.18)		17.21 (−15.35, 49.76)	
*p*-value	0.046		0.395	
Stiffness				
Mean change (SD)	13.93 ± 39.82	−10.53 ± 56.96	7.00 ± 83.43	22.61 ± 70.79
Group difference (95% CI)	24.47 (−12.49, 61.42)		−15.61 (−73.55, 42.33)	
*p*-value	0.093		0.836	
Creep				
Mean change (SD)	−0.10 ± 0.28	0.11 ± 0.29	0.02 ± 0.49	−0.12 ± 0.34
Group difference (95% CI)	−0.21 (−0.42, 0.00)		0.14 (−0.18, 0.45)	
*p*-value	0.056		0.633	
Decrement				
Mean change (SD)	−0.06 ± 0.98	−0.04 ± 0.68	0.12 ± 0.65	−0.12 ± 0.73
Group difference (95% CI)	−0.01 (−0.65, 0.62)		0.24 (−0.28, 0.76)	
*p*-value	0.678		0.300	

CI, confidence interval; N-PS, non-paretic side; PS, paretic side; SD, standard deviation. Mean difference denotes (R-BoT + plus − Control) for the change scores; *p*-values are from between-group comparisons.

## Data Availability

The data presented in this study are not publicly available due to privacy and ethical restrictions. All relevant data are accessible to the corresponding author and may be provided upon reasonable request.

## Supplementary Materials

The following supporting information can be downloaded at: https://www.mdpi.com/article/10.3390/jcm15072514/s1, Table S1: Additional clinical outcomes: baseline and post-treatment scores and baseline-adjusted between-group differences; Table S2: Sensitivity analyses of between-group differences in change scores using the Wilcoxon rank-sum test; Table S3: Comparison of changes in muscle mechanical properties from baseline to post-treatment between groups, stratified by paretic status (PS vs. N-PS); Table S4: Correlation between MyotonPRO parameters and lower-limb motor function in paretic and non-paretic sides.

## Abbreviations

The following abbreviations are used in this manuscript:

ANCOVA	Analysis of covariance
BRS-LE	Brunnstrom recovery stage of the lower extremities
BRS-UE	Brunnstrom recovery stage of the upper extremities
CI	Confidence interval
FAC	Functional Ambulation Category
FMA-LE	Fugl–Meyer Assessment for the Lower Extremity
IQR	Interquartile range
MAS	Modified Ashworth Scale
MTS	Modified Tardieu Scale
SD	Standard deviation
SE	Standard error
