# Supplementary material for: Effects of Robot-Assisted Gait Training on Stage-Based Lower Limb Motor Recovery and Muscle Tone in Subacute Stroke: A Randomized Controlled Trial
Source: J Clin Med. 2026 Mar 25;15(7):2514. doi: 10.3390/jcm15072514 (PMC13073898; doi:10.3390/jcm15072514)
Supplement: Supplementary file 1 [file jcm-15-02514-s001.zip › Supplementary table_S1-edited.pdf]

Supplementary Table S1. Additional clinical outcomes: baseline and post-treatment scores and baseline-adjusted between-group differences.

Outcome	Group	Pre	Post	Adjusted Group difference†	p-value	Effect size (Hedges' g)‡
**Motor Recovery**						
BRS lower limb (primary)	R-BoT+plus	2.43 ± 1.60	4.50 ± 1.34	1.09 (0.24, 1.94)	0.014	0.99
	Control	2.60 ± 1.59	3.53 ± 1.73			
BRS upper limb	R-BoT+plus	2.36 ± 1.55	3.79 ± 2.01	0.61 (-0.40, 1.62)	0.228	0.49
	Control	2.53 ± 1.68	3.33 ± 1.76			
**Gait Function**						
FAC	R-BoT+plus	0.93 ± 0.70	2.60 ± 1.72	0.81 (0.03, 1.59)	0.041	0.87
	Control	0.67 ± 0.72	1.33 ± 1.40			
**K-FMA Scores**						
Lower limb	R-BoT+plus	14.31 ± 7.49	18.08 ± 8.36	-0.41 (-4.60, 3.77)	0.840	0.15
	Control	12.50 ± 11.41	17.07 ± 9.60			
Coordination/speed	R-BoT+plus	1.92 ± 1.71	2.54 ± 2.11	-0.27 (-1.52, 0.98)	0.655	0.20
	Control	1.57 ± 2.17	2.50 ± 2.50			
Motor function (lower+coord)	R-BoT+plus	16.23 ± 8.48	20.62 ± 9.95	-0.74 (-5.60, 4.13)	0.758	0.18
	Control	14.07 ± 13.25	19.57 ± 11.60			
Sensation	R-BoT+plus	6.92 ± 3.97	9.31 ± 3.28	0.89 (-1.32, 3.11)	0.413	0.10
	Control	5.36 ± 5.23	7.43 ± 4.47			
Passive joint motion	R-BoT+plus	19.23 ± 2.77	20.00 ± 0.00	0.97 (-0.51, 2.44)	0.188	0.19
	Control	15.86 ± 7.46	17.50 ± 5.69			
Joint pain	R-BoT+plus	19.08 ± 2.75	19.77 ± 0.44	0.91 (-0.73, 2.55)	0.262	0.31
	Control	14.93 ± 8.27	17.21 ± 5.77			
**Spasticity (MAS)**						
Elbow flexors	R-BoT+plus	0.36 ± 0.63	0.64 ± 0.84	0.09 (-0.56, 0.73)	0.787	0.25
	Control	0.60 ± 0.74	0.67 ± 0.82			

Wrist flexors	R-BoT+plus	0.43 ± 0.94	0.43 ± 0.76	-0.08 (-0.76, 0.60)	0.808	0.08
	Control	0.47 ± 0.64	0.53 ± 1.06			
Hip adductors	R-BoT+plus	0.43 ± 0.76	0.43 ± 0.65	0.01 (-0.55, 0.57)	0.972	0.16
	Control	0.67 ± 0.72	0.53 ± 0.92			
Knee extensors	R-BoT+plus	0.07 ± 0.27	0.07 ± 0.27	-0.08 (-0.34, 0.18)	0.524	0.13
	Control	0.27 ± 0.46	0.33 ± 0.82			
Ankle plantar flexors	R-BoT+plus	0.29 ± 0.73	0.43 ± 0.76	-0.04 (-0.64, 0.57)	0.906	0.25
	Control	0.73 ± 1.33	0.60 ± 0.91			
**Spasticity (MTS, R2-R1)**						
Elbow flexors	R-BoT+plus	13.57 ± 27.63	7.50 ± 11.89	-6.96 (-22.20, 8.27)	0.356	0.40
	Control	9.00 ± 11.98	13.67 ± 24.53			
Elbow extensors	R-BoT+plus	6.07 ± 12.12	17.86 ± 31.67	4.73 (-19.24, 28.69)	0.689	0.13
	Control	5.33 ± 15.52	13.00 ± 23.59			
Wrist flexors	R-BoT+plus	5.71 ± 18.69	5.71 ± 13.99	0.45 (-13.86, 14.76)	0.949	0.16
	Control	10.00 ± 17.32	7.00 ± 18.11			
Wrist extensors	R-BoT+plus	0.00 ± 0.00	1.07 ± 2.89	1.07 (-0.58, 2.72)	0.194	0.55
	Control	3.33 ± 10.47	0.00 ± 0.00			
Hip abductors	R-BoT+plus	4.64 ± 12.16	0.71 ± 2.67	0.02 (-0.49, 0.52)	0.945	0.44
	Control	0.67 ± 2.58	0.00 ± 0.00			
Hip adductors	R-BoT+plus	2.14 ± 5.79	8.93 ± 17.34	6.83 (-7.17, 20.84)	0.325	0.70
	Control	7.67 ± 7.76	4.67 ± 8.55			
Knee flexors	R-BoT+plus	0.71 ± 2.67	8.57 ± 24.05	6.22 (-7.91, 20.35)	0.374	0.53
	Control	6.67 ± 15.55	4.00 ± 8.28			
Knee extensors	R-BoT+plus	0.00 ± 0.00	0.00 ± 0.00	-1.59 (-5.10, 1.92)	0.361	NE
	Control	1.67 ± 3.62	1.67 ± 5.23			
Ankle dorsiflexors	R-BoT+plus	3.57 ± 9.29	4.64 ± 9.09	1.83 (-4.94, 8.60)	0.584	0.28
	Control	5.33 ± 11.25	3.33 ± 7.24			

Ankle plantarflexors	R-BoT+plus	0.00 ± 0.00	0.00 ± 0.00	NE	NE	NE
	Control	1.33 ± 5.16	2.00 ± 7.75			

BRS-LE, Brunnstrom recovery stage for the lower extremities; BRS-UE, Brunnstrom recovery stage for the upper extremities; FAC, Functional Ambulation Category; CI, confidence interval; SD, standard deviation.

Values are presented as means ± SD.

Not estimable (NE) indicates that the statistic could not be reliably estimated due to zero variability (e.g., no change in one group) and/or model non-convergence/singularity for the ANCOVA.

†Adjusted difference indicates the baseline-adjusted between-group difference (R-BoT+plus minus Control) estimated from ANCOVA with post-treatment score as the outcome, group as the factor of interest, and baseline score as a covariate. HC3 heteroscedasticity-robust standard errors were used to derive 95% CIs and p-values.

‡ Effect sizes were quantified using Hedges' g based on change scores (Post–Pre).
