# Supplementary material for: Effects of Robot-Assisted Gait Training on Stage-Based Lower Limb Motor Recovery and Muscle Tone in Subacute Stroke: A Randomized Controlled Trial
Source: J Clin Med. 2026 Mar 25;15(7):2514. doi: 10.3390/jcm15072514 (PMC13073898; doi:10.3390/jcm15072514)
Supplement: Supplementary file 1 [file jcm-15-02514-s001.zip › Supplementary table_S2-edited.pdf]

Supplementary Table S2. Sensitivity analyses of between-group differences in change scores using the Wilcoxon rank-sum test.

Outcome	HL difference (R-bot+plus–Control), 95% CI	p-value
**Motor Recovery**		
BRS lower limb (primary)	1.00 (0.00, 2.00)	0.004
BRS upper limb	1.00 (0.00, 1.00)	0.069
**Gait Function**		
FAC	1.00 (0.00, 2.00)	0.035
**K-FMA Scores**		
Lower limb	-1.00 (-5.00, 4.00)	0.714
Coordination/speed	0.00 (-1.00, 1.00)	0.897
Motor function (lower+coord)	0.93 (-6.00, 4.00)	0.751
Sensation	0.00 (-2.00, 2.00)	0.839
Passive joint motion	0.00 (0.00, 0.00)	0.694
Joint pain	0.00 (-1.00, 0.00)	0.354
**Spasticity (MAS)**		
Elbow flexors	0.00 (0.00, 1.00)	0.540
Wrist flexors	0.00 (0.00, 0.00)	0.598
Hip adductors	0.00 (0.00, 1.00)	0.493
Knee extensors	0.00 (0.00, 0.00)	1.000
Ankle plantar flexors	0.00 (0.00, 0.00)	0.959
**Spasticity (MTS, R2-R1)**		
Elbow flexors	0.00 (-20.00, 10.00)	0.892
Elbow extensors	0.00 (-10.00, 10.00)	0.615
Wrist flexors	0.00 (0.00, 10.00)	0.116
Wrist extensors	0.00 (0.00, 0.00)	0.054
Hip abductors	0.00 (0.00, 0.00)	0.483
Hip adductors	5.00 (0.00, 20.00)	0.034
Knee flexors	0.00 (0.00, 10.00)	0.189
Knee extensors	0.00 (0.00, 0.00)	0.326
Ankle dorsiflexors	0.00 (0.00, 0.00)	1.000
Ankle plantarflexors	0.00 (0.00, 10.00)	0.365

Values are reported as Hodges–Lehmann (HL) estimators with 95% confidence intervals (CIs). Between-group differences were assessed using the Wilcoxon rank-sum test on change scores (Δ = Post–Pre).
HL differences are presented as R-BoT+plus minus Control.
