# Supplementary material for: Effects of Robot-Assisted Gait Training on Stage-Based Lower Limb Motor Recovery and Muscle Tone in Subacute Stroke: A Randomized Controlled Trial
Source: J Clin Med. 2026 Mar 25;15(7):2514. doi: 10.3390/jcm15072514 (PMC13073898; doi:10.3390/jcm15072514)
Supplement: Supplementary file 1 [file jcm-15-02514-s001.zip › Supplementary table_S3-edited.pdf]

Supplementary table S3. Comparison of changes in muscle mechanical properties from baseline to post-treatment between groups, stratified by paretic status (PS vs. N-PS).

Classification	Group	Pre	Post	Change (Post-Pre)†	Group difference‡, 95% CI	Effect Size
**Biceps Contraction Creep**						
PS	R-BoT+plus group	1.41 ± 0.24	1.42 ± 0.19	0.01 ± 0.26	0.04 (-0.15, 0.23)	0.14
	Control group	1.41 ± 0.24	1.39 ± 0.29	-0.02 ± 0.25		
N-PS	R-BoT+plus group	1.30 ± 0.22	1.36 ± 0.13	0.06 ± 0.25	0.06 (-0.10, 0.22)	0.29
	Control group	1.43 ± 0.31	1.43 ± 0.28	-0.00 ± 0.15		
**Biceps Contraction Decrement**						
PS	R-BoT+plus group	1.42 ± 0.20	1.49 ± 0.68	0.07 ± 0.63	-0.18 (-0.55, 0.19)	0.36
	Control group	1.39 ± 0.25	1.28 ± 0.26	-0.11 ± 0.25		
N-PS	R-BoT+plus group	1.52 ± 0.39	1.37 ± 0.23	-0.15 ± 0.32	0.04 (-0.16, 0.25)	0.15
	Control group	1.37 ± 0.31	1.26 ± 0.24	-0.11 ± 0.22		
**Biceps Contraction Frequency (Hz)**						
PS	R-BoT+plus group	13.11 ± 1.91	12.79 ± 2.05	-0.31 ± 1.38	-0.27 (-1.39, 0.85)	0.17
	Control group	12.71 ± 1.81	12.67 ± 1.95	-0.05 ± 1.60		
N-PS	R-BoT+plus group	13.47 ± 1.82	13.03 ± 0.64	-0.43 ± 1.69	-0.63 (-1.86, 0.60)	0.38
	Control group	12.45 ± 2.10	12.65 ± 1.82	0.20 ± 1.59		
**Biceps Contraction Relaxation (ms)**						
PS	R-BoT+plus group	22.13 ± 3.59	22.94 ± 3.32	0.81 ± 2.29	1.06 (-1.37, 3.49)	0.32
	Control group	23.05 ± 4.01	22.81 ± 4.96	-0.25 ± 3.93		
N-PS	R-BoT+plus group	21.23 ± 3.74	22.20 ± 1.98	0.97 ± 4.16	0.90 (-1.70, 3.50)	0.25
	Control group	23.22 ± 5.27	23.29 ± 4.60	0.07 ± 2.55		
**Biceps Contraction Stiffness (N/m)**						
PS	R-BoT+plus group	229.87 ± 51.10	208.40 ± 54.82	-21.47 ± 35.91*	-16.80 (-47.21, 13.61)	0.4
	Control group	213.93 ± 46.76	209.27 ± 48.46	-4.67 ± 44.75		

N-PS	R-BoT+plus group	237.53 ± 59.85	221.13 ± 27.48	-16.40 ± 59.07	-9.07 (-48.29, 30.16)	0.17
	Control group	218.07 ± 53.05	210.73 ± 48.96	-7.33 ± 44.41		
**Biceps femoris Contraction Creep**						
PS	R-BoT+plus group	1.26 ± 0.22	1.27 ± 0.24	0.00 ± 0.22	-0.03 (-0.27, 0.21)	0.09
	Control group	1.31 ± 0.32	1.34 ± 0.29	0.03 ± 0.40		
N-PS	R-BoT+plus group	1.32 ± 0.22	1.22 ± 0.25	-0.10 ± 0.25	-0.01 (-0.29, 0.26)	0.03
	Control group	1.33 ± 0.28	1.24 ± 0.30	-0.09 ± 0.45		
**Biceps femoris Contraction Decrement**						
PS	R-BoT+plus group	1.80 ± 0.51	1.74 ± 0.44	-0.07 ± 0.44	0.03 (-0.34, 0.39)	0.05
	Control group	2.02 ± 0.37	1.93 ± 0.55	-0.09 ± 0.52		
N-PS	R-BoT+plus group	1.77 ± 0.44	1.82 ± 0.56	0.04 ± 0.56	0.08 (-0.26, 0.42)	0.17
	Control group	1.86 ± 0.43	1.82 ± 0.41	-0.04 ± 0.31		
**Biceps femoris Contraction Frequency (Hz)**						
PS	R-BoT+plus group	14.05 ± 2.20	14.06 ± 2.79	0.01 ± 1.96	-0.53 (-2.41, 1.36)	0.2
	Control group	12.89 ± 1.92	13.43 ± 3.39	0.54 ± 2.95		
N-PS	R-BoT+plus group	13.65 ± 2.06	14.61 ± 2.96	0.96 ± 2.47	0.23 (-2.18, 2.63)	0.07
	Control group	13.45 ± 2.37	14.19 ± 2.57	0.73 ± 3.78		
**Biceps femoris Contraction Relaxation (ms)**						
PS	R-BoT+plus group	20.20 ± 3.46	20.22 ± 3.99	0.02 ± 3.60	-0.35 (-4.40, 3.70)	0.06
	Control group	20.77 ± 5.49	21.14 ± 4.87	0.37 ± 6.64		
N-PS	R-BoT+plus group	21.11 ± 3.58	19.46 ± 4.12	-1.65 ± 4.25	-0.27 (-4.70, 4.16)	0.05
	Control group	21.20 ± 4.50	19.82 ± 5.02	-1.38 ± 7.12		
**Biceps femoris Contraction Stiffness (N/m)**						
PS	R-BoT+plus group	268.13 ± 50.81	269.67 ± 69.08	1.53 ± 58.65	-4.27 (-47.52, 38.98)	0.07
	Control group	255.00 ± 57.48	260.80 ± 69.96	5.80 ± 56.99		
N-PS	R-BoT+plus group	264.73 ± 54.62	288.67 ± 76.29	23.93 ± 79.56	13.07 (-49.07, 75.20)	0.15
	Control group	263.40 ± 62.42	274.27 ± 66.71	10.87 ± 86.39		

**Biceps femoris Relaxation Creep**						
PS	R-BoT+plus group	1.68 ± 0.35	1.57 ± 0.35	-0.10 ± 0.28	-0.21 (-0.42, 0.00)	0.72
	Control group	1.66 ± 0.30	1.76 ± 0.39	0.11 ± 0.29		
N-PS	R-BoT+plus group	1.65 ± 0.38	1.67 ± 0.35	0.02 ± 0.49	0.14 (-0.18, 0.45)	0.32
	Control group	1.59 ± 0.27	1.48 ± 0.37	-0.12 ± 0.34		
**Biceps femoris Relaxation Decrement**						
PS	R-BoT+plus group	2.17 ± 0.77	2.11 ± 0.46	-0.06 ± 0.98	-0.01 (-0.65, 0.62)	0.02
	Control group	2.33 ± 0.64	2.28 ± 0.58	-0.04 ± 0.68		
N-PS	R-BoT+plus group	2.13 ± 0.68	2.26 ± 0.61	0.12 ± 0.65	0.24 (-0.28, 0.76)	0.34
	Control group	2.27 ± 0.60	2.15 ± 0.69	-0.12 ± 0.73		
**Biceps femoris Relaxation Frequency (Hz)**						
PS	R-BoT+plus group	10.14 ± 2.10	11.40 ± 2.08	1.26 ± 2.27	1.91 (0.29, 3.53)	0.86
	Control group	10.85 ± 2.17	10.20 ± 1.93	-0.65 ± 2.05		
N-PS	R-BoT+plus group	10.79 ± 1.65	11.21 ± 1.95	0.42 ± 2.52	9.69 (-11.57, 30.96)	0.35
	Control group	20.70 ± 38.83	11.43 ± 2.40	-9.27 ± 38.34		
**Biceps femoris Relaxation Relaxation (ms)**						
PS	R-BoT+plus group	26.93 ± 5.92	25.38 ± 5.75	-1.55 ± 4.52	-3.31 (-6.79, 0.18)	0.69
	Control group	26.55 ± 4.96	28.31 ± 6.52	1.75 ± 4.79		
N-PS	R-BoT+plus group	26.64 ± 6.44	26.69 ± 5.70	0.69 ± 7.91	17.21 (-15.35, 49.76)	0.39
	Control group	40.46 ± 57.86	23.94 ± 5.94	-16.52 ± 58.42		
**Biceps femoris Relaxation Stiffness (N/m)**						
PS	R-BoT+plus group	189.33 ± 62.59	203.27 ± 66.67	13.93 ± 39.82	24.47 (-12.49, 61.42)	0.48
	Control group	194.87 ± 59.00	184.33 ± 69.79	-10.53 ± 56.96		
N-PS	R-BoT+plus group	188.40 ± 59.68	195.40 ± 61.69	7.00 ± 83.43	-15.61 (-73.55, 42.33)	0.2
	Control group	189.99 ± 72.10	212.60 ± 72.05	22.61 ± 70.79		
**Biceps Relaxation Creep**						
PS	R-BoT+plus group	1.54 ± 0.22	1.53 ± 0.23	-0.02 ± 0.27	-0.13 (-0.32, 0.05)	0.53

	Control group	1.48 ± 0.27	1.60 ± 0.32	0.12 ± 0.22		
N-PS	R-BoT+plus group	1.53 ± 0.22	1.58 ± 0.18	0.06 ± 0.21	0.06 (-0.07, 0.19)	0.33
	Control group	1.53 ± 0.26	1.53 ± 0.20	-0.00 ± 0.11		
**Biceps Relaxation Decrement**						
PS	R-BoT+plus group	1.57 ± 0.26	1.53 ± 0.24	-0.04 ± 0.28	-0.03 (-0.26, 0.21)	0.08
	Control group	1.70 ± 0.28	1.69 ± 0.25	-0.01 ± 0.34		
N-PS	R-BoT+plus group	1.61 ± 0.33	1.50 ± 0.22	-0.11 ± 0.34	-0.22 (-0.49, 0.04)	0.61
	Control group	1.62 ± 0.30	1.73 ± 0.39	0.11 ± 0.37		
**Biceps Relaxation Frequency (Hz)**						
PS	R-BoT+plus group	11.18 ± 1.23	11.39 ± 1.32	0.21 ± 1.76	0.69 (-0.50, 1.88)	0.42
	Control group	11.63 ± 1.55	11.15 ± 1.66	-0.48 ± 1.40		
N-PS	R-BoT+plus group	11.36 ± 1.78	11.17 ± 0.92	-0.19 ± 1.64	0.09 (-0.93, 1.10)	0.06
	Control group	11.40 ± 1.52	11.13 ± 1.32	-0.27 ± 0.95		
**Biceps Relaxation Relaxation (ms)**						
PS	R-BoT+plus group	25.01 ± 3.53	24.79 ± 3.82	-0.22 ± 4.42	-2.11 (-5.07, 0.84)	0.52
	Control group	24.15 ± 4.58	26.05 ± 5.48	1.89 ± 3.38		
N-PS	R-BoT+plus group	24.83 ± 3.86	25.68 ± 2.89	0.85 ± 3.50	1.62 (-0.94, 4.18)	0.46
	Control group	25.35 ± 6.19	24.58 ± 3.49	-0.77 ± 3.34		
**Biceps Relaxation Stiffness (N/m)**						
PS	R-BoT+plus group	191.93 ± 43.01	190.87 ± 53.00	-1.07 ± 62.18	13.60 (-24.75, 51.95)	0.26
	Control group	202.40 ± 43.02	187.73 ± 50.01	-14.67 ± 35.67		
N-PS	R-BoT+plus group	191.00 ± 50.39	178.80 ± 28.68	-12.20 ± 53.28	-9.27 (-40.70, 22.16)	0.22
	Control group	193.80 ± 42.18	190.87 ± 40.21	-2.93 ± 23.42		
**Gastrocnemius Contraction Creep**						
PS	R-BoT+plus group	1.12 ± 0.24	1.12 ± 0.24	-0.00 ± 0.33	0.09 (-0.16, 0.33)	0.26
	Control group	1.12 ± 0.29	1.03 ± 0.28	-0.09 ± 0.32		
N-PS	R-BoT+plus group	1.08 ± 0.27	1.07 ± 0.26	-0.01 ± 0.26	0.02 (-0.15, 0.20)	0.1

	Control group	1.03 ± 0.16	1.00 ± 0.18	-0.04 ± 0.21		
**Gastrocnemius Contraction Decrement**						
PS	R-BoT+plus group	1.36 ± 0.34	1.27 ± 0.26	-0.09 ± 0.33	-0.16 (-0.37, 0.06)	0.54
	Control group	1.42 ± 0.29	1.49 ± 0.33	0.07 ± 0.23		
N-PS	R-BoT+plus group	1.34 ± 0.32	1.41 ± 0.33	0.07 ± 0.33	0.16 (-0.22, 0.53)	0.31
	Control group	1.46 ± 0.59	1.37 ± 0.22	-0.09 ± 0.62		
**Gastrocnemius Contraction Frequency (Hz)**						
PS	R-BoT+plus group	15.37 ± 2.91	15.31 ± 1.69	-0.07 ± 3.47	-0.84 (-3.25, 1.57)	0.25
	Control group	15.71 ± 3.24	16.48 ± 3.12	0.77 ± 2.94		
N-PS	R-BoT+plus group	16.35 ± 3.83	15.82 ± 2.54	-0.53 ± 3.29	-1.26 (-3.60, 1.08)	0.39
	Control group	16.17 ± 2.64	16.90 ± 2.48	0.73 ± 2.96		
**Gastrocnemius Contraction Relaxation (ms)**						
PS	R-BoT+plus group	18.03 ± 3.99	17.85 ± 3.80	-0.18 ± 5.40	1.35 (-2.60, 5.29)	0.25
	Control group	17.92 ± 4.65	16.39 ± 4.44	-1.53 ± 5.14		
N-PS	R-BoT+plus group	17.21 ± 4.63	17.00 ± 4.43	-0.21 ± 4.17	-10.81 (-35.46, 13.83)	0.33
	Control group	16.56 ± 2.59	27.17 ± 44.59	10.61 ± 44.37		
**Gastrocnemius Contraction Stiffness (N/m)**						
PS	R-BoT+plus group	300.00 ± 74.62	304.73 ± 64.79	4.73 ± 93.48	-21.47 (-90.12, 47.19)	0.23
	Control group	313.60 ± 85.63	339.80 ± 77.88	26.20 ± 90.05		
N-PS	R-BoT+plus group	334.07 ± 128.65	336.47 ± 121.59	2.40 ± 93.64	-22.67 (-95.10, 49.77)	0.23
	Control group	320.33 ± 57.04	345.40 ± 87.51	25.07 ± 99.91		
**Gastrocnemius Relaxation Creep**						
PS	R-BoT+plus group	1.56 ± 0.28	1.62 ± 0.23	0.06 ± 0.26	0.17 (-0.06, 0.41)	0.53
	Control group	1.65 ± 0.34	1.54 ± 0.37	-0.11 ± 0.36		
N-PS	R-BoT+plus group	1.56 ± 0.22	1.58 ± 0.20	0.02 ± 0.23	-0.02 (-0.19, 0.15)	0.08
	Control group	1.54 ± 0.22	1.58 ± 0.16	0.04 ± 0.23		

**Gastrocnemius Relaxation Decrement**						
PS	R-BoT+plus group	1.82 ± 0.39	1.84 ± 0.57	0.02 ± 0.61	0.10 (-0.33, 0.53)	0.17
	Control group	2.05 ± 0.49	1.97 ± 0.61	-0.08 ± 0.54		
N-PS	R-BoT+plus group	1.83 ± 0.46	1.82 ± 0.43	-0.01 ± 0.28	0.82 (-1.44, 3.09)	0.29
	Control group	2.70 ± 3.64	1.92 ± 0.40	-0.83 ± 3.92		
**Gastrocnemius Relaxation Frequency (Hz)**						
PS	R-BoT+plus group	12.15 ± 2.15	11.76 ± 1.63	-0.39 ± 1.81	-1.39 (-2.80, 0.01)	0.72
	Control group	11.16 ± 1.96	12.16 ± 1.70	1.00 ± 1.94		
N-PS	R-BoT+plus group	20.41 ± 33.67	11.83 ± 1.43	-8.57 ± 33.48	-8.83 (-27.39, 9.72)	0.36
	Control group	11.59 ± 1.24	11.85 ± 0.97	0.26 ± 1.52		
Gastrocnemius Relaxation Relaxation (ms)						
PS	R-BoT+plus group	24.77 ± 4.44	25.67 ± 3.67	0.90 ± 4.15	2.61 (-1.10, 6.31)	0.51
	Control group	26.25 ± 5.15	24.55 ± 5.94	-1.71 ± 5.61		
N-PS	R-BoT+plus group	24.93 ± 3.57	25.17 ± 3.22	0.25 ± 3.63	-0.23 (-2.94, 2.49)	0.06
	Control group	24.73 ± 3.36	25.20 ± 2.62	0.47 ± 3.64		
**Gastrocnemius Relaxation Stiffness (N/m)**						
PS	R-BoT+plus group	221.40 ± 43.34	209.27 ± 41.54	-12.13 ± 37.39	-28.53 (-67.11, 10.04)	0.54
	Control group	207.47 ± 36.27	223.87 ± 61.38	16.40 ± 61.79		
N-PS	R-BoT+plus group	212.40 ± 36.41	207.53 ± 37.99	-4.87 ± 41.72	-3.07 (-35.75, 29.61)	0.07
	Control group	203.07 ± 37.18	201.27 ± 34.31	-1.80 ± 45.55		
**Rectus femoris Contraction Creep**						
PS	R-BoT+plus group	1.00 ± 0.20	0.98 ± 0.14	-0.01 ± 0.15	0.03 (-0.13, 0.18)	0.13
	Control group	1.12 ± 0.22	1.08 ± 0.26	-0.04 ± 0.24		
N-PS	R-BoT+plus group	1.01 ± 0.24	1.01 ± 0.13	-0.00 ± 0.21	0.02 (-0.13, 0.17)	0.1
	Control group	1.03 ± 0.24	1.01 ± 0.22	-0.02 ± 0.18		
**Rectus femoris Contraction Decrement**						
PS	R-BoT+plus group	1.40 ± 0.43	1.34 ± 0.24	-0.07 ± 0.35	-0.03 (-0.24, 0.18)	0.11

	Control group	1.48 ± 0.29	1.45 ± 0.25	-0.03 ± 0.17		
N-PS	R-BoT+plus group	1.43 ± 0.32	1.37 ± 0.28	-0.06 ± 0.28	-0.10 (-0.36, 0.15)	0.3
	Control group	1.47 ± 0.18	1.51 ± 0.37	0.04 ± 0.38		
**Rectus femoris Contraction Frequency (Hz)**						
PS	R-BoT+plus group	17.20 ± 3.26	17.05 ± 2.10	-0.15 ± 3.13	-1.07 (-4.40, 2.25)	0.24
	Control group	14.83 ± 4.63	15.75 ± 3.31	0.92 ± 5.38		
N-PS	R-BoT+plus group	16.95 ± 2.90	17.01 ± 1.80	0.05 ± 2.14	0.54 (-1.11, 2.19)	0.24
	Control group	16.72 ± 2.63	16.23 ± 2.40	-0.49 ± 2.28		
**Rectus femoris Contraction Relaxation (ms)**						
PS	R-BoT+plus group	15.72 ± 3.29	15.55 ± 2.29	-0.17 ± 2.50	0.29 (-2.19, 2.78)	0.09
	Control group	17.69 ± 3.56	17.22 ± 4.36	-0.47 ± 3.93		
N-PS	R-BoT+plus group	15.96 ± 3.80	16.00 ± 2.00	0.04 ± 3.26	0.25 (-2.10, 2.61)	0.08
	Control group	16.31 ± 3.90	16.10 ± 3.86	-0.21 ± 3.03		
**Rectus femoris Contraction Stiffness (N/m)**						
PS	R-BoT+plus group	363.87 ± 92.92	352.20 ± 49.94	-11.67 ± 79.42	-15.00 (-76.65, 46.65)	0.18
	Control group	325.47 ± 70.53	328.80 ± 91.73	3.33 ± 85.28		
N-PS	R-BoT+plus group	357.20 ± 92.39	348.93 ± 37.62	-8.27 ± 80.29	-3.40 (-58.85, 52.05)	0.04
	Control group	352.33 ± 74.96	347.47 ± 75.33	-4.87 ± 67.20		
**Rectus femoris Relaxation Creep**						
PS	R-BoT+plus group	1.28 ± 0.25	1.28 ± 0.24	-0.01 ± 0.20	0.07 (-0.08, 0.22)	0.32
	Control group	1.21 ± 0.30	1.13 ± 0.29	-0.07 ± 0.20		
N-PS	R-BoT+plus group	1.33 ± 0.30	1.25 ± 0.21	-0.08 ± 0.23	0.04 (-0.13, 0.22)	0.19
	Control group	1.25 ± 0.32	1.13 ± 0.30	-0.12 ± 0.23		
**Rectus femoris Relaxation Decrement**						
PS	R-BoT+plus group	1.65 ± 0.44	1.86 ± 0.75	0.20 ± 0.54	0.43 (-0.04, 0.89)	0.67
	Control group	1.93 ± 0.51	1.71 ± 0.47	-0.23 ± 0.69		
N-PS	R-BoT+plus group	1.77 ± 0.64	1.88 ± 0.52	0.11 ± 0.44	0.46 (0.12, 0.80)	0.99

	Control group	1.91 ± 0.49	1.56 ± 0.27	-0.35 ± 0.47*		
**Rectus femoris Relaxation Frequency (Hz)**						
PS	R-BoT+plus group	13.35 ± 1.98	13.14 ± 2.24	-0.21 ± 1.92	-0.75 (-2.58, 1.08)	0.3
	Control group	13.61 ± 2.56	14.15 ± 2.40	0.54 ± 2.86		
N-PS	R-BoT+plus group	12.73 ± 1.94	13.41 ± 1.59	0.68 ± 1.72	-0.98 (-2.79, 0.83)	0.41
	Control group	13.10 ± 2.17	14.72 ± 2.59	1.66 ± 2.80		
**Rectus femoris Relaxation Relaxation (ms)**						
PS	R-BoT+plus group	20.42 ± 4.07	20.34 ± 3.97	-0.08 ± 3.22	1.03 (-1.39, 3.46)	0.31
	Control group	19.25 ± 5.03	18.13 ± 4.83	-1.11 ± 3.25		
N-PS	R-BoT+plus group	21.15 ± 4.90	19.98 ± 3.51	-1.17 ± 3.81	0.69 (-2.17, 3.56)	0.18
	Control group	19.91 ± 5.38	18.05 ± 4.92	-1.87 ± 3.84		
**Rectus femoris Relaxation Stiffness (N/m)**						
PS	R-BoT+plus group	238.89 ± 75.42	270.53 ± 64.42	31.64 ± 105.59	21.65 (-46.13, 89.43)	0.23
	Control group	284.13 ± 79.47	294.13 ± 96.47	9.99 ± 71.19		
N-PS	R-BoT+plus group	262.93 ± 81.64	275.87 ± 54.63	12.93 ± 67.68	-17.80 (-68.77, 33.17)	0.25
	Control group	274.27 ± 65.67	305.00 ± 79.20	30.73 ± 68.59		
**Tibialis ant Contraction (Dorsiflexion) Creep**						
PS	R-BoT+plus group	1.14 ± 0.14	1.14 ± 0.17	-0.01 ± 0.15	0.04 (-0.16, 0.24)	0.14
	Control group	1.22 ± 0.30	1.17 ± 0.26	-0.04 ± 0.34		
N-PS	R-BoT+plus group	1.16 ± 0.19	1.11 ± 0.13	-0.05 ± 0.25	-0.04 (-0.23, 0.16)	0.15
	Control group	1.12 ± 0.22	1.11 ± 0.21	-0.01 ± 0.27		
**Tibialis ant Contraction (Dorsiflexion) Decrement**						
PS	R-BoT+plus group	1.30 ± 0.40	1.23 ± 0.23	-0.06 ± 0.39	-0.06 (-0.36, 0.25)	0.13
	Control group	1.51 ± 0.35	1.50 ± 0.44	-0.01 ± 0.43		
N-PS	R-BoT+plus group	1.28 ± 0.39	1.25 ± 0.24	-0.03 ± 0.47	-0.00 (-0.39, 0.38)	0.01
	Control group	1.47 ± 0.49	1.45 ± 0.39	-0.02 ± 0.55		
**Tibialis ant Contraction (Dorsiflexion) Frequency (Hz)**						

PS	R-BoT+plus group	15.64 ± 1.82	15.88 ± 1.96	0.24 ± 2.26	0.15 (-1.49, 1.80)	0.07
	Control group	14.42 ± 2.02	14.51 ± 2.16	0.09 ± 2.14		
N-PS	R-BoT+plus group	15.74 ± 2.10	16.30 ± 1.80	0.56 ± 1.92	0.28 (-2.34, 2.90)	0.08
	Control group	15.53 ± 2.77	15.81 ± 3.17	0.28 ± 4.45		
**Tibialis ant Contraction (Dorsiflexion) Relaxation (ms)**						
PS	R-BoT+plus group	17.92 ± 2.36	17.93 ± 2.78	0.01 ± 2.39	0.69 (-2.53, 3.90)	0.16
	Control group	19.12 ± 4.99	18.45 ± 4.22	-0.67 ± 5.45		
N-PS	R-BoT+plus group	18.33 ± 2.99	17.49 ± 2.10	-0.83 ± 3.92	-1.68 (-5.35, 2.00)	0.33
	Control group	16.66 ± 5.41	17.51 ± 3.33	0.84 ± 5.70		
**Tibialis ant Contraction (Dorsiflexion) Stiffness (N/m)**						
PS	R-BoT+plus group	320.20 ± 54.65	317.53 ± 62.97	-2.67 ± 53.43	-9.67 (-60.99, 41.66)	0.14
	Control group	312.13 ± 71.98	319.13 ± 69.06	7.00 ± 80.23		
N-PS	R-BoT+plus group	319.80 ± 51.81	328.87 ± 38.53	9.07 ± 64.44	2.87 (-53.41, 59.15)	0.04
	Control group	323.13 ± 64.54	329.33 ± 65.41	6.20 ± 84.26		
**Tibialis ant Contraction (Plantarflexion) Creep**						
PS	R-BoT+plus group	0.97 ± 0.13	0.90 ± 0.17	-0.07 ± 0.18	-0.05 (-0.20, 0.11)	0.21
	Control group	1.01 ± 0.32	0.99 ± 0.26	-0.02 ± 0.24		
N-PS	R-BoT+plus group	0.98 ± 0.18	0.81 ± 0.09	-0.16 ± 0.21**	-0.18 (-0.35, -0.01)	0.79
	Control group	0.92 ± 0.19	0.94 ± 0.18	0.02 ± 0.24		
**Tibialis ant Contraction (Plantarflexion) Decrement**						
PS	R-BoT+plus group	1.19 ± 0.25	1.20 ± 0.23	0.01 ± 0.24	0.10 (-0.13, 0.34)	0.33
	Control group	1.39 ± 0.34	1.30 ± 0.40	-0.09 ± 0.37		
N-PS	R-BoT+plus group	1.14 ± 0.28	1.13 ± 0.12	-0.01 ± 0.31	-0.02 (-0.24, 0.19)	0.07
	Control group	1.22 ± 0.26	1.23 ± 0.24	0.01 ± 0.26		
**Tibialis ant Contraction (Plantarflexion) Frequency (Hz)**						
PS	R-BoT+plus group	17.51 ± 1.96	18.93 ± 2.95	1.42 ± 2.96	1.15 (-0.94, 3.25)	0.4
	Control group	16.97 ± 3.46	17.24 ± 3.26	0.27 ± 2.63		

N-PS	R-BoT+plus group	125.91 ± 417.48	20.50 ± 2.46	-105.41 ± 417.24	-104.83 (-335.90, 126.23)	0.35
	Control group	18.57 ± 3.37	17.99 ± 2.88	-0.58 ± 4.13		
**Tibialis ant Contraction (Plantarflexion) Relaxation (ms)**						
PS	R-BoT+plus group	15.49 ± 2.48	14.13 ± 2.97	-1.36 ± 2.89	-1.16 (-3.80, 1.48)	0.32
	Control group	15.97 ± 5.27	15.77 ± 3.94	-0.20 ± 4.05		
N-PS	R-BoT+plus group	15.20 ± 2.42	12.73 ± 1.48	-2.47 ± 2.95**	-2.78 (-5.37, -0.19)	0.78
	Control group	14.48 ± 3.06	14.79 ± 2.94	0.31 ± 3.88		
**Tibialis ant Contraction (Plantarflexion) Stiffness (N/m)**						
PS	R-BoT+plus group	369.73 ± 58.13	409.40 ± 88.92	39.67 ± 81.11	28.57 (-52.15, 109.30)	0.26
	Control group	357.04 ± 135.78	368.13 ± 65.65	11.09 ± 127.82		
N-PS	R-BoT+plus group	373.07 ± 56.46	441.33 ± 56.20	68.27 ± 79.13**	76.27 (6.48, 146.05)	0.8
	Control group	398.67 ± 87.10	390.67 ± 69.75	-8.00 ± 105.02		
**Tibialis ant Relaxation Creep**						
PS	R-BoT+plus group	1.13 ± 0.21	1.03 ± 0.16	-0.10 ± 0.25	-0.10 (-0.27, 0.08)	0.4
	Control group	1.13 ± 0.30	1.13 ± 0.24	-0.01 ± 0.23		
N-PS	R-BoT+plus group	1.09 ± 0.12	0.98 ± 0.14	-0.11 ± 0.24	-0.11 (-0.26, 0.04)	0.52
	Control group	1.05 ± 0.21	1.05 ± 0.19	-0.00 ± 0.16		
**Tibialis ant Relaxation Decrement**						
PS	R-BoT+plus group	1.31 ± 0.34	1.23 ± 0.23	-0.08 ± 0.29	-0.01 (-0.27, 0.26)	0.01
	Control group	1.51 ± 0.31	1.43 ± 0.39	-0.08 ± 0.40		
N-PS	R-BoT+plus group	1.26 ± 0.37	1.23 ± 0.33	-0.03 ± 0.48	0.04 (-0.27, 0.36)	0.1
	Control group	1.41 ± 0.37	1.34 ± 0.22	-0.07 ± 0.34		
**Tibialis ant Relaxation Frequency (Hz)**						
PS	R-BoT+plus group	15.52 ± 1.97	16.83 ± 2.42	1.31 ± 2.94	1.08 (-0.75, 2.91)	0.43
	Control group	14.90 ± 1.80	15.13 ± 2.15	0.23 ± 1.78		
N-PS	R-BoT+plus group	16.08 ± 1.87	17.46 ± 2.05	1.38 ± 2.50	1.81 (0.07, 3.54)	0.76

	Control group	16.59 ± 2.61	16.16 ± 2.22	-0.43 ± 2.10		
**Tibialis ant Relaxation Relaxation (ms)**						
PS	R-BoT+plus group	17.81 ± 3.44	16.21 ± 2.70	-1.59 ± 4.03	-1.48 (-4.39, 1.43)	0.37
	Control group	17.90 ± 4.96	17.79 ± 3.92	-0.11 ± 3.75		
N-PS	R-BoT+plus group	17.27 ± 2.13	15.40 ± 2.13	-1.87 ± 3.95	-1.91 (-4.36, 0.54)	0.57
	Control group	16.58 ± 3.31	16.62 ± 3.03	0.04 ± 2.32		
**Tibialis ant Relaxation Stiffness (N/m)**						
PS	R-BoT+plus group	324.87 ± 59.79	350.47 ± 69.84	25.60 ± 74.80	24.40 (-27.64, 76.44)	0.34
	Control group	328.20 ± 76.35	329.40 ± 67.21	1.20 ± 63.75		
N-PS	R-BoT+plus group	328.60 ± 47.78	370.33 ± 37.39	41.73 ± 76.82	61.97 (-12.16, 136.11)	0.61
	Control group	340.51 ± 107.59	320.27 ± 63.31	-20.24 ± 116.09		

†Within-group changes (Post–Pre) and corresponding 95% confidence intervals were estimated using the Wilcoxon signed-rank test (Hodges–Lehmann estimator for paired differences).

‡The between-group difference (R-BoT+plus - Control) and corresponding 95% confidence intervals were estimated using the Wilcoxon rank-sum test (Hodges–Lehmann estimator).

* $p < .05$, ** $p < .01$, *** $p < .001$.
