# Supplementary material for: Effects of Robot-Assisted Gait Training on Stage-Based Lower Limb Motor Recovery and Muscle Tone in Subacute Stroke: A Randomized Controlled Trial
Source: J Clin Med. 2026 Mar 25;15(7):2514. doi: 10.3390/jcm15072514 (PMC13073898; doi:10.3390/jcm15072514)
Supplement: Supplementary file 1 [file jcm-15-02514-s001.zip › Supplementary table_S4-edited.pdf]

Supplementary Table S4. Correlation between MyotonPRO parameters and lower-limb motor function in paretic and non-paretic sides.

Parameters	PS		N-PS	
	ρ	p-value	ρ	p-value
**Biceps femoris – contraction**				
creep	0.268	0.355	-0.095	0.748
decrement	-0.131	0.654	0.383	0.177
frequency	-0.37	0.194	0.037	0.900
relaxation	0.318	0.267	-0.095	0.747
stiffness	-0.334	0.242	0.044	0.882
**Biceps femoris – relaxation**				
creep	0.507	0.064	-0.224	0.442
decrement	0.252	0.385	-0.238	0.413
frequency	-0.343	0.230	0.012	0.969
relaxation	0.519	0.057	-0.25	0.410
stiffness	-0.173	0.554	-0.039	0.894
**Biceps – contraction**				
creep	-0.443	0.112	-0.328	0.253
decrement	-0.256	0.376	-0.001	0.997
frequency	0.632	0.015*	0.275	0.342
relaxation	-0.454	0.103	-0.399	0.157
stiffness	0.554	0.048*	0.379	0.182
**Biceps – relaxation**				
creep	-0.265	0.359	-0.002	0.994
decrement	-0.127	0.666	0.145	0.620
frequency	0.227	0.436	0.058	0.845
relaxation	-0.253	0.383	-0.002	0.994
stiffness	0.202	0.488	-0.131	0.654
**Gastrocnemius – contraction**				
creep	0.369	0.194	0.291	0.313
decrement	-0.132	0.654	-0.514	0.060
frequency	-0.131	0.654	0.021	0.944
relaxation	0.321	0.264	0.316	0.271
stiffness	-0.104	0.724	-0.33	0.249
**Gastrocnemius – relaxation**				
creep	0.251	0.386	0.686	0.007**
decrement	0.64	0.014*	0.337	0.238
frequency	-0.423	0.132	-0.66	0.010*
relaxation	0.212	0.466	0.677	0.008**
stiffness	0.113	0.700	-0.648	0.012*
**Rectus femoris – contraction**				

creep	-0.647	0.012*	-0.464	0.095
decrement	0.178	0.544	-0.166	0.570
frequency	0.391	0.166	0.447	0.109
relaxation	-0.657	0.011*	-0.464	0.095
stiffness	0.381	0.179	0.492	0.074
**Rectus femoris – relaxation**				
creep	-0.173	0.554	0.159	0.587
decrement	-0.364	0.200	0.042	0.888
frequency	0.388	0.171	-0.211	0.468
relaxation	-0.263	0.363	0.159	0.587
stiffness	-0.089	0.762	-0.097	0.742
**Tibialis ant – contraction (dorsiflexion)**				
creep	-0.065	0.826	-0.088	0.766
decrement	0.21	0.471	0.323	0.260
frequency	-0.408	0.147	-0.215	0.461
relaxation	-0.042	0.888	-0.088	0.766
stiffness	0.01	0.972	0.127	0.666
**Tibialis ant – contraction (plantarflexion)**				
creep	-0.109	0.712	-0.397	0.160
decrement	-0.04	0.891	0.348	0.222
frequency	0.078	0.790	0.576	0.031*
relaxation	-0.234	0.420	-0.459	0.099
stiffness	0.173	0.554	0.392	0.165
**Tibialis ant – relaxation**				
creep	-0.282	0.329	-0.161	0.581
decrement	0.21	0.471	0.365	0.200
frequency	-0.062	0.832	0.164	0.576
relaxation	-0.249	0.390	-0.212	0.466
stiffness	0.261	0.368	0.226	0.437
